# Modern Medical Therapeutics

**Published:** 1885-03

**Authors:** 


MODERN MEDICAL THERAPEUTICS.—A Compendium of recent formulæ and specific therapeutical directions, from the practice of eminent contemporary physicians, American and Foreign. By George H. Napheys, A. M., M. D. Edited by Joseph F. Edwards, M. D., and D. G. Brinton, M. D. Eighth edition; enlarged and revised. Philadelphia: D. G. Brinton, 115 S. Seventh street. 1885.

This eminently practical treatise is so well and favorably known to the medical public through the large editions which have been previously published, that we have only to draw attention to the new matter incorporated in the present issue.

The recent additions to the materia medica, large in number, and some of great importance, have been introduced and their merits stated, and a portion of this new matter has never before been published.

Those who would adopt the standard methods of eminent physicians in treating diseases, will find many of their most approved prescriptions for special affections incorporated in this work, and it may be consulted with profit.

